# Supplementary figures and images for: Transcriptional regulatory divergence underpinning species-specific learned vocalization in songbirds
Source: PLoS Biol. 2019 Nov 13;17(11):e3000476. doi: 10.1371/journal.pbio.3000476 (PMC6853299; doi:10.1371/journal.pbio.3000476)

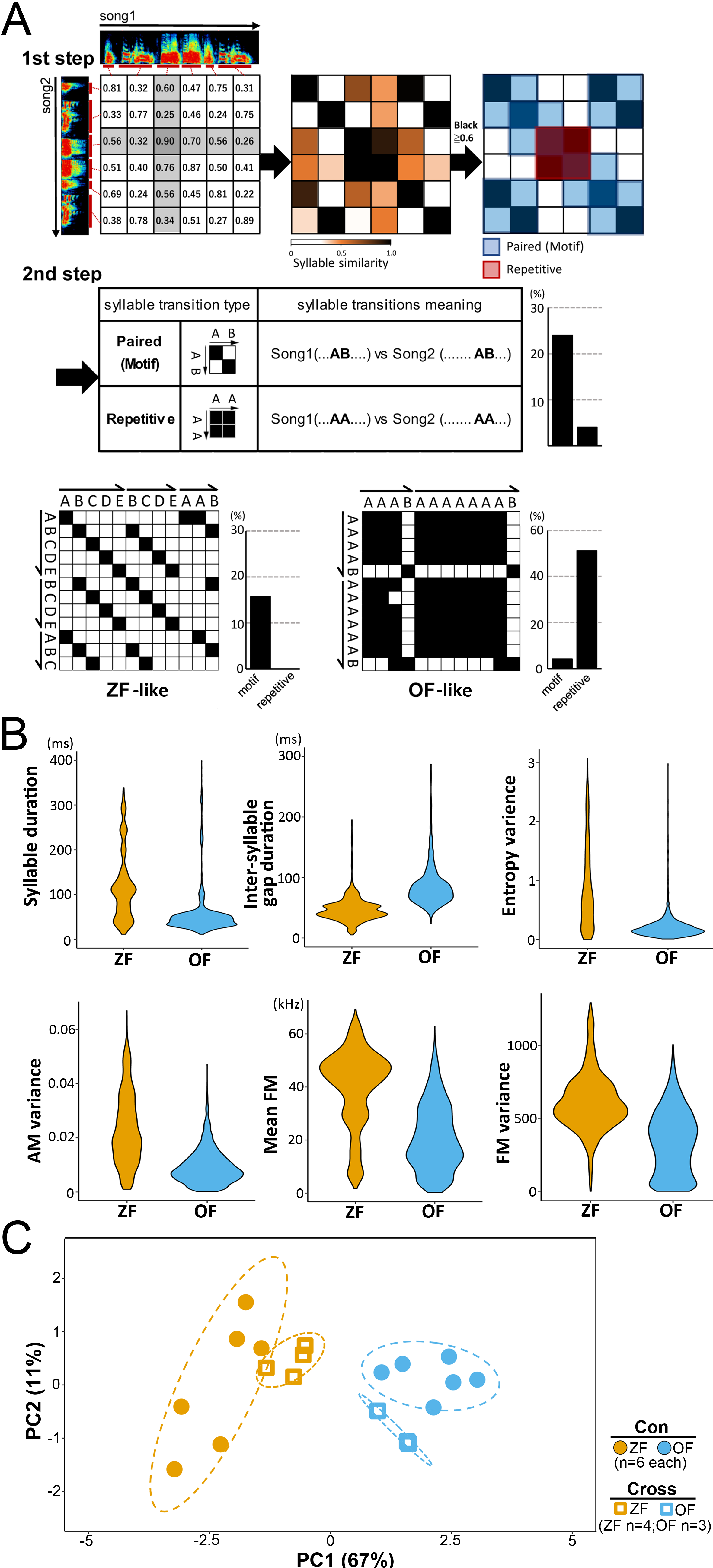

Supplement: S1 Fig — (A) (Upper panels) SSM analysis for the detection of syllable sequential transition patterns. The SSM comprises two steps: First, a correlation matrix including the syllable similarity scores was prepared using the round-robin comparison of all syllables in two songs to maintain the sequential order of the syllables in the songs. These similarity scores in the matrix were binarized at a threshold at 0.595. Second, the occurrence rate of two patterns of binarized “2 row × 2 column” cells in the SSM was calculated as a percentage of the paired (motif) and repetitive-syllable transition types (see the Materials and methods). (Lower panel) Test examples of the SSM method using artificial song models mimicking the songs with motif and repetitive sequences. (B) The similar distribution range of syllable acoustic traits between ZF and OF. Violin plots of the distribution of syllable duration, inter-syllable gap duration, entropy variance, AM variance, mean FM, and FM variance from ZF and OF that were reared with conspecific song tutoring (total 3,000 syllables from n = 6 birds each and 500 syllables/bird). (C) PCA of the song features of ZFs and OFs reared under conspecific and cross-species song tutoring conditions (“Con”: n = 6 each from conspecific song tutored ZF and OF; “Cross”: n = 4 and 3 from cross-species song tutored ZF and OF, respectively). Relevant data values are included in S1 Data for panels B and C. AM, amplitude modulation; FM, frequency modulation; OF, owl finch; PCA, principal component analysis; SSM, syllable similarity matrix; ZF, zebra finch. (TIF) [file pbio.3000476.s001.tif]

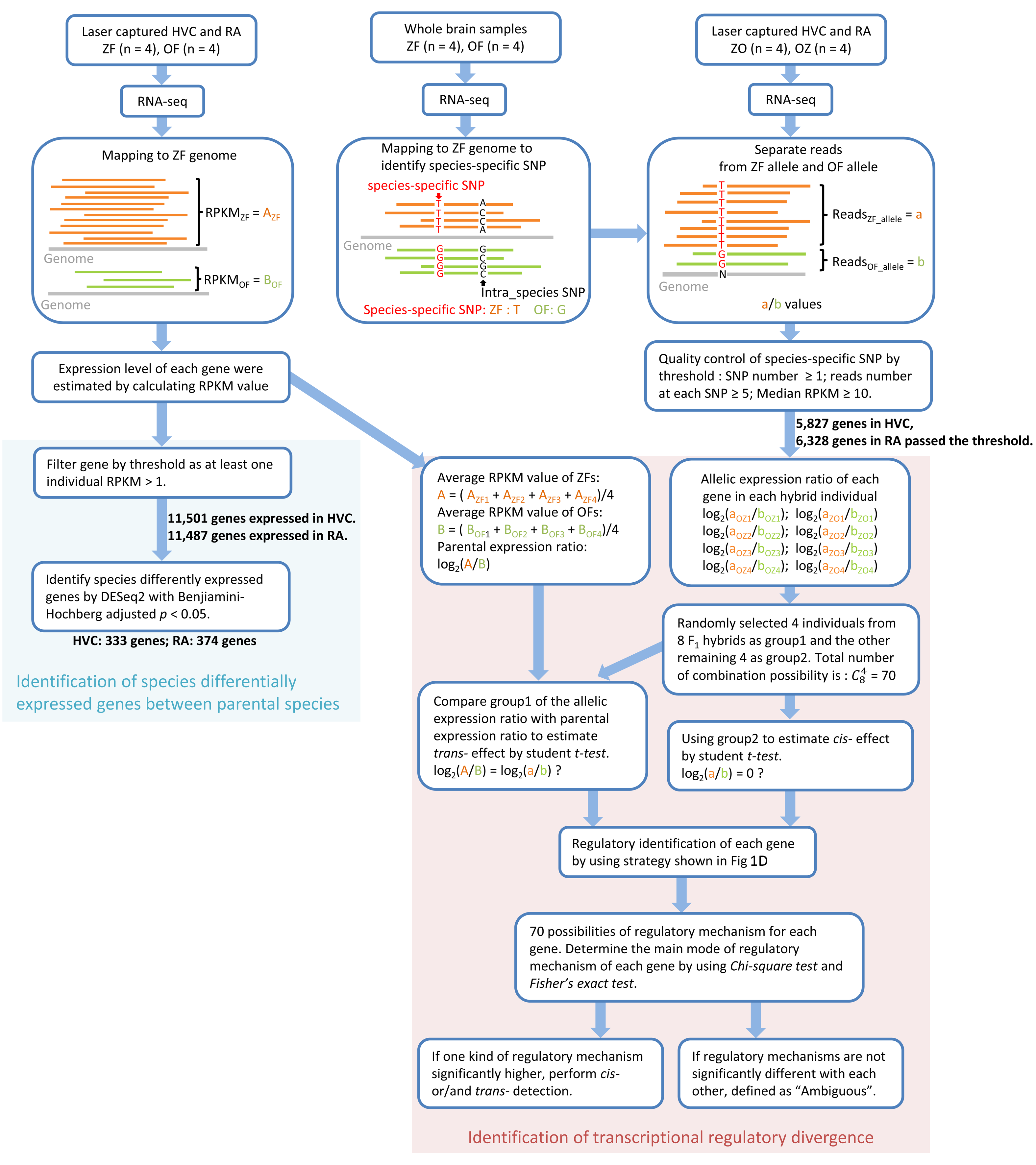

Supplement: S2 Fig — (TIF) [file pbio.3000476.s002.tif]

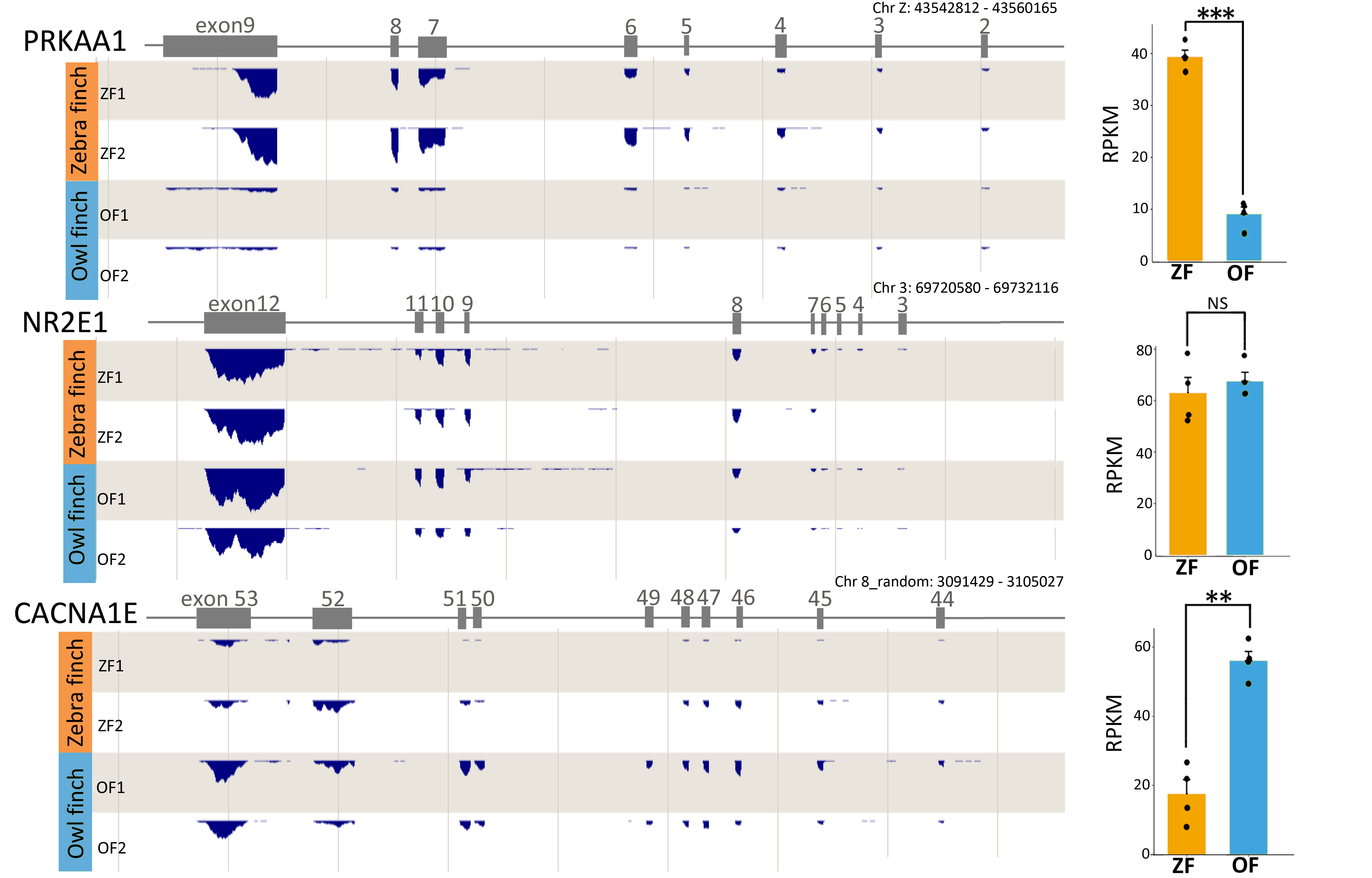

Supplement: S3 Fig — (Left panels) Expression levels of PRKAA1, NR2E1, and CACNA1E in song nucleus HVC of ZFs and OFs. Gray-colored boxes represent the position of exons for each gene. Dark blue peaks below exons represent read density. (Right panels) Gene expression levels in ZF and OF. Each dot represents RPKM value for the individual. Mean ± SEM (n = 4 birds each, one-way ANOVA, *p < 0.05; n.s., not significant). Relevant data values are included in S2 Data. CACNA1E, calcium voltage-gated channel subunit alpha 1E; NR2E1, nuclear receptor subfamily 2 group E member 1; OF, owl finch; PRKAA1, protein kinase AMP-activated catalytic subunit alpha 1; RPKM, reads per kilobase of transcript per million reads mapped; ZF, zebra finch. (TIF) [file pbio.3000476.s003.tif]

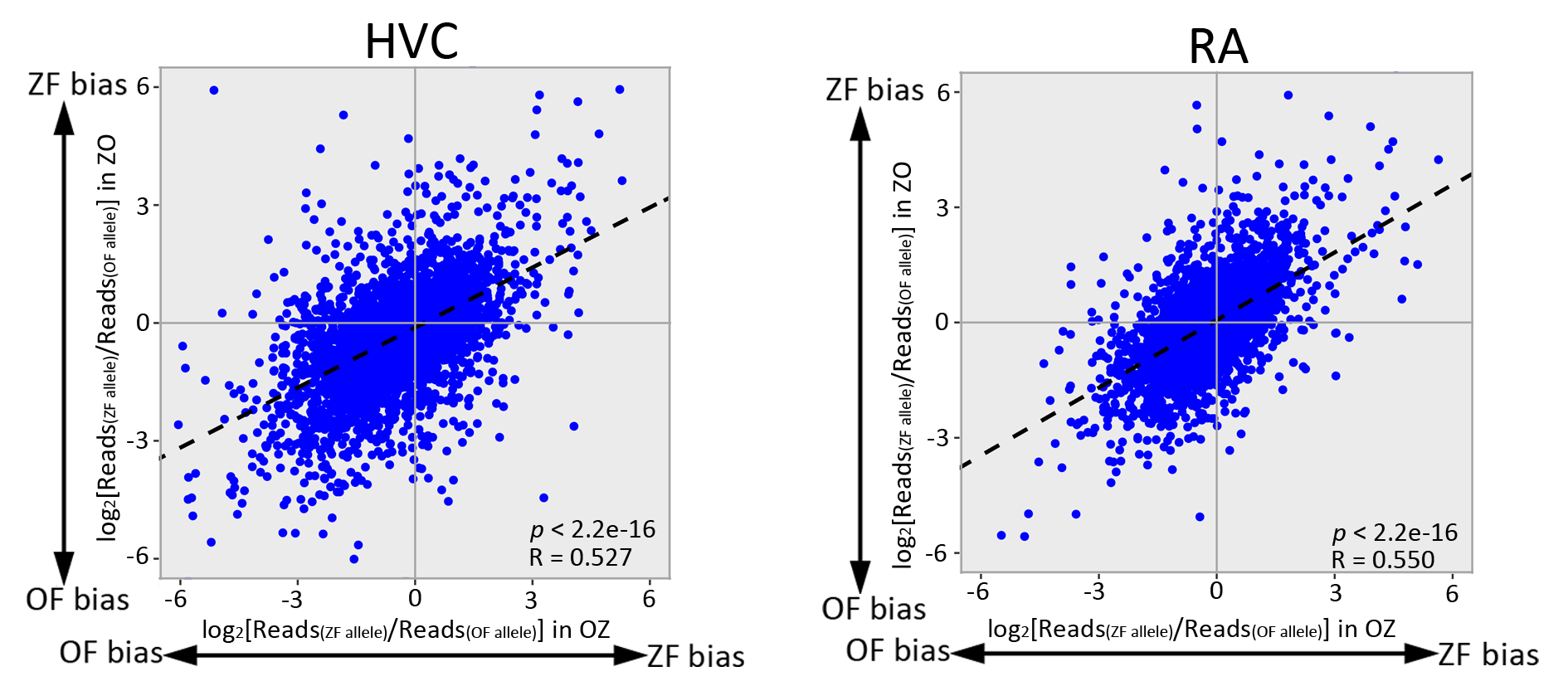

Supplement: S4 Fig — Scatterplots of allelic expression ratios of 5,849 and 6,328 genes in HVC and RA, respectively, of OZ and ZO hybrids (Spearman correlation coefficient). Relevant data values are included in S3 Data. F1, first-generation; OF, owl finch; OZ, F1 hybrid offspring between OF♀ and ZF♂; RA, robust nucleus of the arcopallium; ZF, zebra finch; ZO, F1 hybrid offspring between ZF♀ and OF♂. (TIF) [file pbio.3000476.s004.tif]

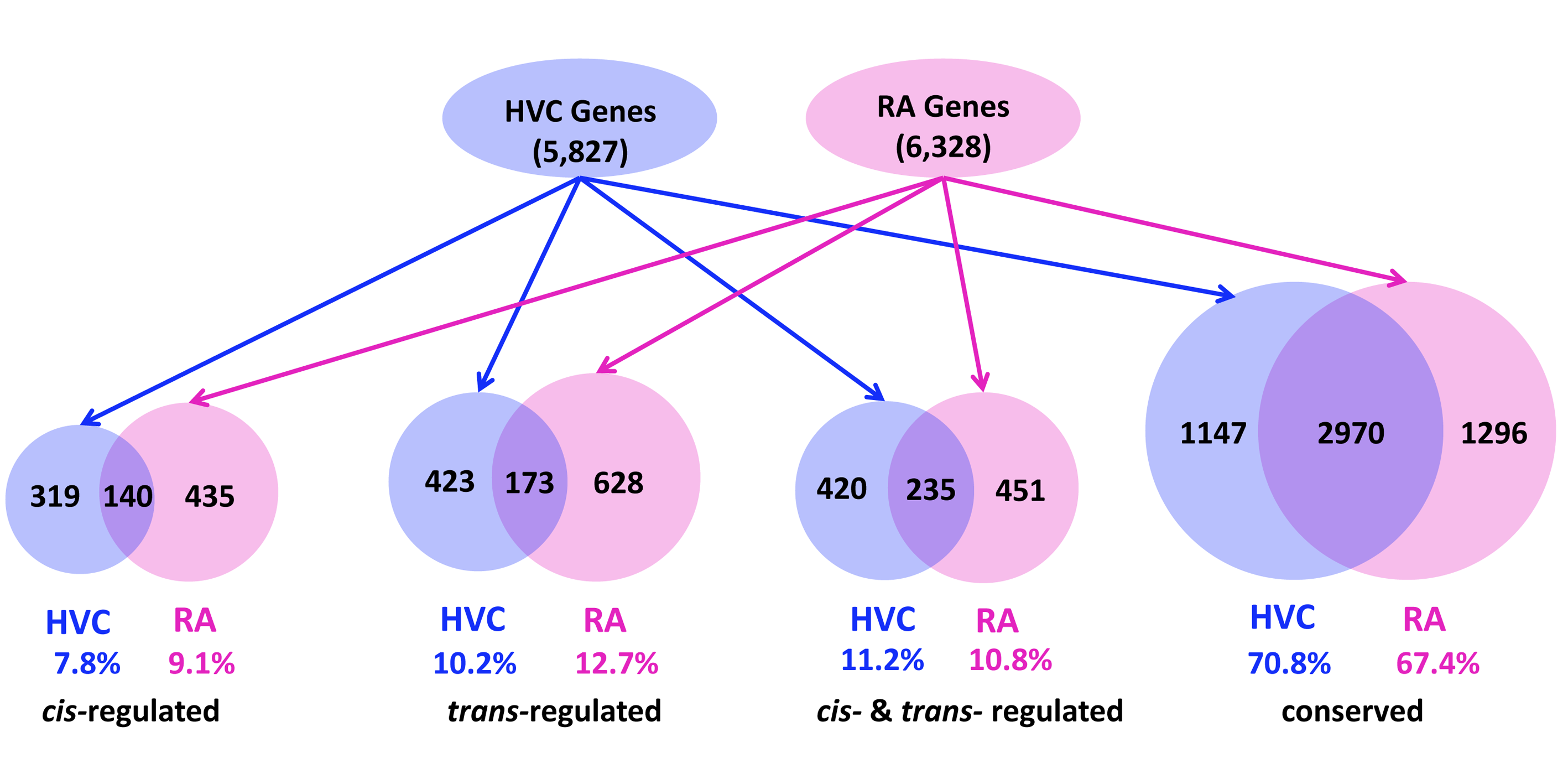

Supplement: S5 Fig — Relevant data values are included in S3 Data. ASE, allele-specific expression; F1, first-generation; RA, robust nucleus of the arcopallium. (TIF) [file pbio.3000476.s005.tif]

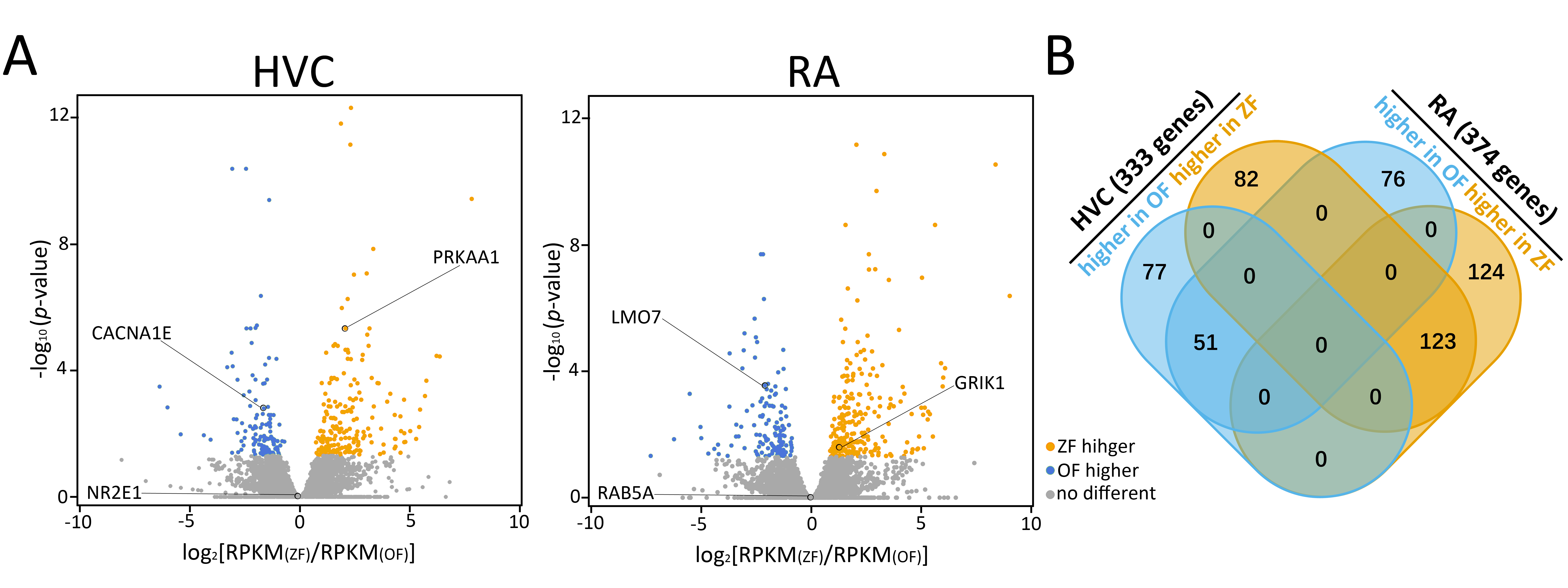

Supplement: S6 Fig — (A) SDE genes in HVC and RA. Orange- and blue-colored spots represent significantly higher expression in ZF or OF, respectively (DEseq2 corrected with the Benjamini-Hochberg method, p < 0.05). (B) Venn diagram representing the number of genes in HVC and RA that are differently expressed between ZF or OF. Relevant data values are included in S3 Data. OF, owl finch; RA, robust nucleus of the arcopallium; SDE, species-differentially expressed; ZF, zebra finch. (TIF) [file pbio.3000476.s006.tif]

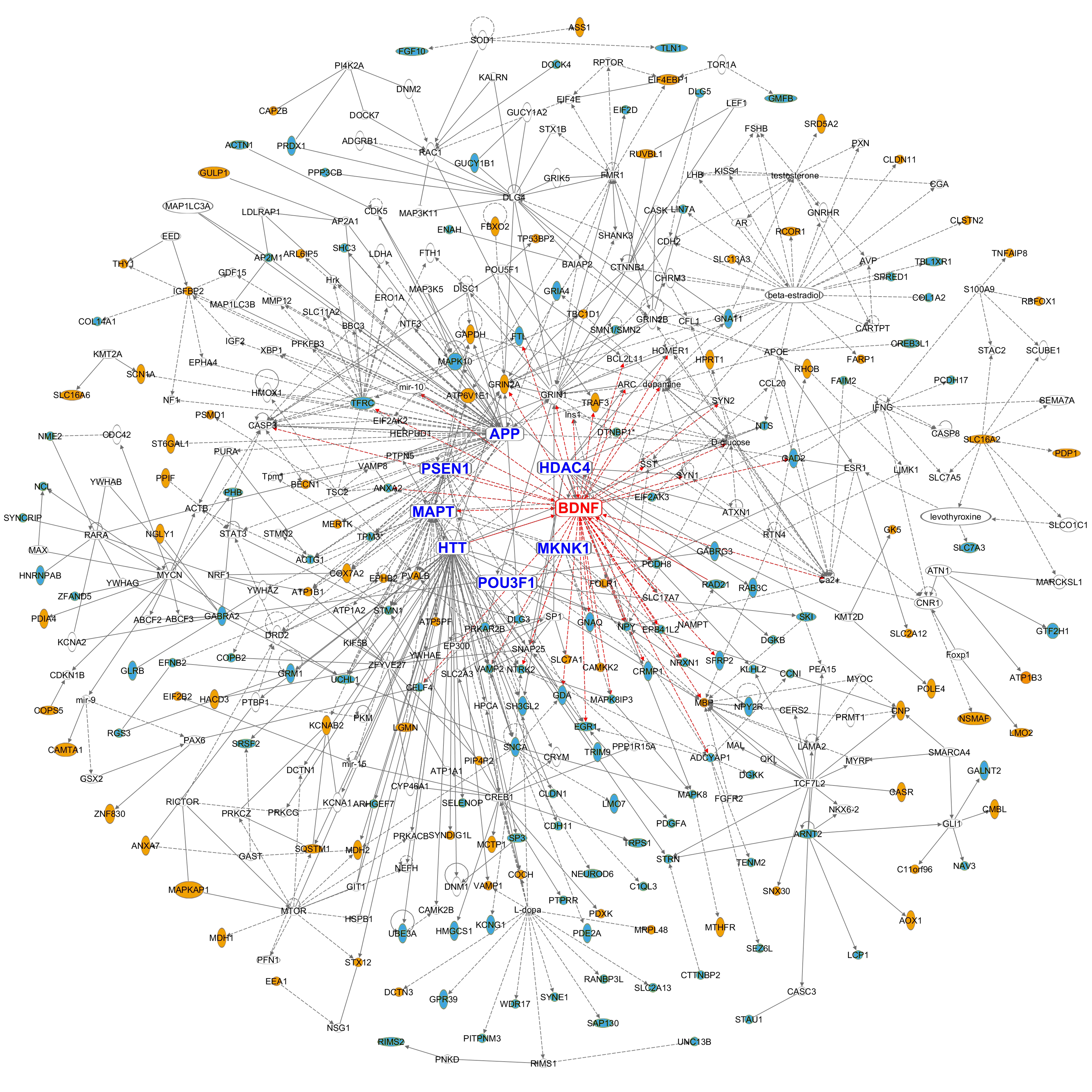

Supplement: S7 Fig — Top 7 candidate upstream mediators, including BDNF, HTT, POU3F1, MAPT, MNKK1, PSEN1, and HDAC4. Trans-regulated genes by BDNF in RA are noted in red. Orange- and green-colored genes are trans-regulated genes that are significantly expressed more highly in RA of ZF or OF, respectively. Relevant data values are included in S4 Data. BDNF, brain-derived neurotrophic factor; HDAC4, histone deacetylase 4; HTT, huntingtin; MAPT, microtubule-associated protein tau; MNKK1, MAP kinase-interacting serine/threonine protein kinase 1; OF, owl finch; POU3F1, POU class 3 homeobox 1; PSEN1, presenilin 1; RA, robust nucleus of the arcopallium; ZF, zebra finch. (TIF) [file pbio.3000476.s007.tif]

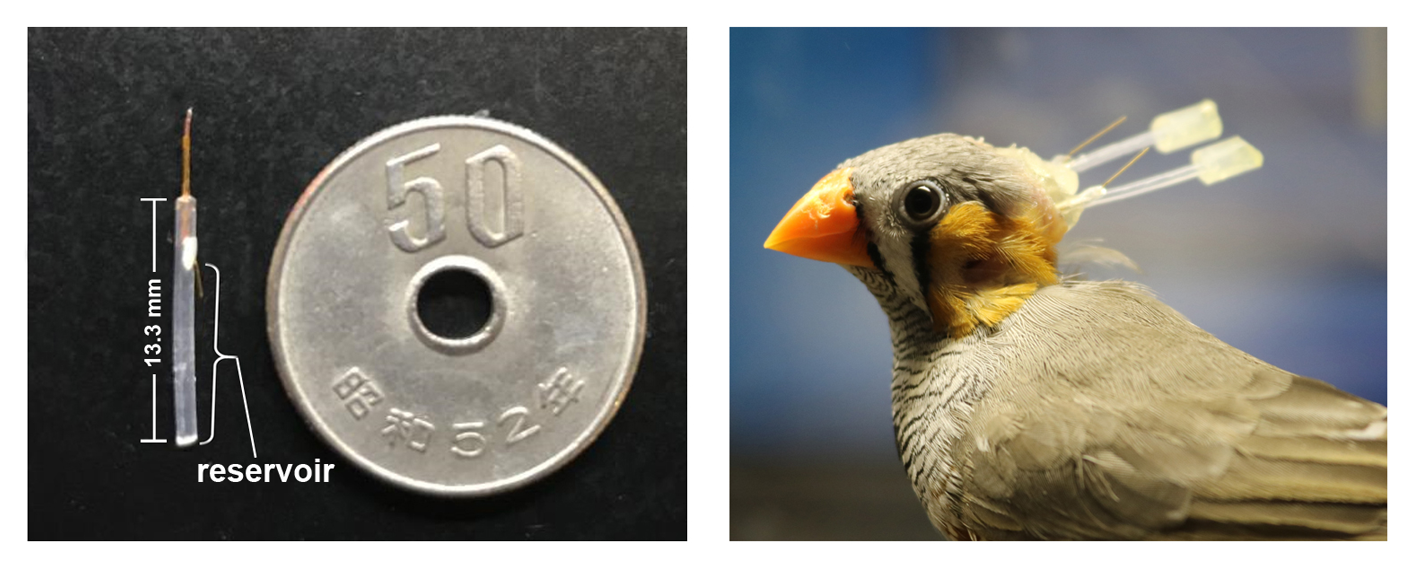

Supplement: S8 Fig — (Left) Photograph of homemade microdialysis probe. (Right) A ZF with microdialysis probes bilaterally implanted in RA. BDNF, brain-derived neurotrophic factor; RA, robust nucleus of the arcopallium; ZF, zebra finch. (TIF) [file pbio.3000476.s008.tif]
