# Supplementary material for: Transcriptional regulatory divergence underpinning species-specific learned vocalization in songbirds
Source: PLoS Biol. 2019 Nov 13;17(11):e3000476. doi: 10.1371/journal.pbio.3000476 (PMC6853299; doi:10.1371/journal.pbio.3000476)
Supplement: Attachment — Submitted filename: PLoS Biology Response to 1st decision v5.2.docx [file pbio.3000476.s015.docx]

**Response letter to reviewers’ comments**

**Reviewer #1 (Yisi Zhang, signed review):**

**Reviewer #1 Comment 1**

*In this work, Wang et al. studied the difference between two closely related but behaviorally different songbird species, the zebra finch and the owl finch, of their transcriptional regulation in the singing system. In particular, the authors investigated the cis- and trans-regulation within HVC and RA and found that the trans-regulation was more prevalent in both areas. The cis- and trans-regulations were closely related to the species-differential gene expression, especially the trans-regulated genes in RA mediated by BDNF. The latter was differentially expressed in HVC. The widespread BDNF expression levels in HVC among the F1 hybrids were significantly correlated with some song features. Over-activation of the BDNF receptor in zebra finch RA eliminated the species-specific song features and altered the expression of some genes in RA. This is an interesting and well-designed comparative study elucidating the evolutional variation of transcriptional regulation within the song production system using RNA sequencing and pharmacological manipulation. The analysis is comprehensive with sufficient statistical analysis. The arguments will be strengthened if the following issues can be clarified.*

**Response**

We thank Dr. Zhang for her supportive comments and for recognizing the scientific value of our work. We also appreciate for the reviewer’s comments, which helped improve our manuscript.

**Reviewer #1 Comment 2**

1. *How different are the genomes of these two species? Since the SNPs are mapped to the*

*ZF reference, does this method naturally introduce bias to the detection of ss-SNPs and the estimation of differential expression? Please discuss.*

**Response**

Although the zebra finch’s whole genome sequence is available in a public database, the same information is currently not available for the owl finch. Therefore, it is not possible to know how many species-specific SNPs (ss-SNPs) exist in the entire genome of the two species, including non-transcribed regions.

In this study, ss-SNP detection was performed using whole brain transcriptome information from ZFs and OFs, as described in the Materials and Methods section (2^nd^ paragraph in page 25). The ss-SNPs identified in this study were located in transcribed regions in the genome sequence. We used the ss-SNP information to distinguish the RNA-seq reads of the alleles of the two species in F_1_ hybrids and to estimate the allelic expression ratio. Therefore, the use of ss-SNPs located at the transcribed regions of the genome is necessary and sufficient. Even if the ss-SNP information was available for the whole genome, we speculate that the estimation of differential transcriptional divergence would not have changed significantly.

We considered that the most effective factor to estimate transcriptional divergence would be a cutoff that is set to extract genes for the calculation of the ASE ratio. In this study, we set the cutoff with ≥5 reads at each ss-SNP position and median RPKM ≥10. This cutoff constitutes a stricter setting than those of other published studies (Wang et al. 2016, *PLoS Biol*; Gomes and Civetta 2015, *Sci Rep*). Therefore, if we set lower thresholds to extract ASE genes, the estimated number of genes with different transcriptional divergence would increase. This issue is discussed in the revised manuscript (first paragraph on page 19).

**Reviewer #1 Comment 3**

*2. The authors hypothesized that BDNF regulates the trans-regulated genes in RA through axon terminal secretion from HVC to RA. Although the infusion of 7,8-DHF altered song structure, the change looks a lot like a song degradation as opposed to a shift to OF-like song. The hypothesis will be strengthened if there is a difference in the concentration of RA BDNF protein between the two species and/or the F1 hybrids.*

**Response**

We thank the reviewer for suggesting a different BDNF concentration in the RA of both species. Although we did not test the BDNF protein level, we examined its mRNA expression level in the RA after diurnal singing (3 h undirected singing) by using *in situ* hybridization. We confirmed that BDNF mRNA expression levels were differently regulated in ZF and OF not only in the HVC but also in the RA (consequently higher in OF than ZF as the silent condition). We added this information in the Results sections (first paragraph on page 14) and as in Fig 5F.

**Reviewer #1 Comment 4**

*3. The authors pointed out that there is no difference in the ASE ratios of the ZO and OZ hybrids and treated them as equivalent. In Figure 6, however, there seems to be a clear division in the BDNF expression levels (at least in RA) between the ZO and OZ. I think this is worth more elaboration or discussion, especially it is interesting that there seems to be more influence from the female side (if I follow the labeling convention of figure 1). I am curious to see if there is a global difference in the song features between ZO, OZ, as well as a comparison with the ZF and OF tutor songs.*

**Response**

In F_1_ hybrids, the ASE rations, but not the expression levels, were used to identify the transcriptional regulatory divergence. When we compared the ASE ratio of BDNF in the HVC and RA of ZO and OZ hybrids, no significant differences were observed between the two hybrid groups (p-value = 0.803 and 0.245 for HVC and RA, respectively; Student’s *t*-test). This result is consistent with our previous description of nonsignificant differences in the ASE ratios of ZO and OZ hybrids.

As indicated by the reviewer, we also realized that some genes in the RA, including BDNF, showed significantly different expression levels (not the ASE ratio) between the ZO and OZ hybrids. Although we could not determine the potential biological meaning of such a difference or its significance in terms of behavioral regulation, we speculated that it may be induced by a potential effect of the mitochondrial genome, which is carried from maternal species, because avian species possess the same set of autosomal chromosomes (Fig 1C).

Although we have tutored ZO/OZ hybrids by using several song types as song tutor models in different experiments, no obvious differences were found between ZO and OZ regarding song features.

**Reviewer #1 Comment 5**

*There are some minor issues:*

*-Ln107, “Fig. 2”. Please specify which sub-figures one should look at. Also, it is not very clear what Fig. 2B is showing, and it is very hard for people not in the songbird field to understand the similarity matrices without explanation.*

**Response**

On the basis of the reviewer’s suggestion, we specified the subfigure information of Fig 2 for the related sentences (page 6). We also added an explanatory drawing as Supplementary Figure 1A (i.e., S1 Fig panel A) to help clarify the syllable similarity matrices shown in Fig 2B.

**Reviewer #1 Comment 6**

*-Why these particular acoustic features were selected but not, for example, entropy, pitch or duration variance?*

**Response**

We first tried to identify the acoustic features of the syllables that differed between ZF and OF by focusing on 10 acoustic features: syllable duration, inter-syllable gap duration, mean pitch, pitch goodness, Wiener entropy, entropy variance, mean AM, AM variance, mean FM, and FM variance. Significant differences were observed in the six acoustic parameters which we used in Fig 2C. To clarify this point, we revised the related sentence in the Materials and Methods (third paragraph on page 23).

**Reviewer #1 Comment 7-11**

*-Ln173, “Fig. 4B” should be “4A”.*

*-Ln258, “diurnal singing in HVC neurons” typo.*

*-Ln314, “are commonly regulated the trans-regulatory environment” typo.*

*-Ln402, “OF checked” typo.*

*-Lns901-906, figure #s are not ordered.*

**Response**

We thank the reviewer for pointing out the typos. All errors have been corrected in the revised manuscript.

**Reviewer #2:**

**Reviewer #2 Comment 1**

*This is a well-written paper form an investigator that has done important contributions to the biology of bird song. In this paper they do pioneering work as they try to identify the genetic basis that could account for differences in the types of song produced by different species.*

*The main strength of the paper is that the strategy is novel for songbirds. A similar approach has been used by Hopi Hoekstra for different behaviors in closely related rodent species, but for birds this is clearly the first such study. The experiments are well-performed, and the explanations are clear. My main concern is that similarly to the Hokestra papers, it is not realistic to expect to be able to pinpoint that the differences for complicated behaviors are due to changes in a single gene. Fundamentally, the final interpretation of this type of approach is that these behavioral differences are due to multiple genes interacting in complex ways. Unfortunately, some features of biology cannot be reduced to simple mechanistic models, and this work is a good example of that caveat.*

**Response**

We sincerely thank the reviewer for the supportive comments and for recognizing the scientific value of our work. We also agree with the reviewer’s comment that explaining complex animal behavior by focusing on a single gene (or a few genes) is reductionist. We acknowledge our overinterpretion of the results on BDNF pharmacological manipulation. We considered that such pharmacological manipulation was useful for evaluating the potential causal links of transcriptional regulation between BDNF and the predicted downstream genes in vivo (the result was shown in Fig 7F in the previous manuscript). However, we should not utilize the BDNF in evaluating the potential drug effects on the species specificity of the song structure. Upon recognizing this, we have entirely and carefully revised the related parts for the BDNF pharmacological experiment.

**Reviewer #2 Comment 2**

*My main criticism about the paper is that I do not believe that the interpretation of the BDNF receptor manipulation is correct. Fundamentally, when they inject the BDNF receptor agonist they see a “simplification” of the song of the zebra finch song – the quality of the song is degraded and it becomes less elaborate. This does not mean that it loses “species-specific acoustic and sequence traits”, it simply means that it becomes degraded. Many other experiments have song that different lesions of the song circuit produce simplified songs that are quite stereotypical. A control for this experiment would be to inject into the birds any other type of drug that affects genes that are NOT present in the genes that they identify as being differentially regulated between zebra and owl finches, and see the behavioral effects. For example, if they inject a drug that messes up a biological process not directly relevant to song production (for example, tubulin polymerization), my guess is that they will see a behavioral change very similar to what they observe with the BDNF agonist (note: they should choose a drug that is not directly toxic to the neurons, but it just messes them up in a reversible manner).*

**Response**

After reading this crucial and judicious indication from the reviewer, we realized that we had incorrectly interpreted the results from the BDNF pharmacological manipulation. As mentioned in our response to Comment 1, the main purpose of the BDNF experiment was to examine the potential links at transcriptional regulation between BDNF and the predicted downstream genes in the RA. As a result, we confirmed that the expression of six (but not all) putative downstream *trans*-regulated genes was altered after BDNF pharmacological manipulation. In parallel, during the BDNF experiment, not only the predicted *trans*-downstream genes but also other altered genes could affect song changes after BDNF agonist administration. Therefore, behavioral data pertaining to song changes should be carefully shown and interpreted. Upon the recognition of our overinterpretation, the Results and Discussion sections related to the BDNF pharmacological experiment were carefully revised. Furthermore, we reordered the panels of Fig 7 to show RNA-seq data as the first panel (i.e., Fig 7A) and removed the behavioral data on owl finches to avoid leading potential readers to consider that zebra finches changed their song to match those of the owl finch after the infusion of the BDNF agonist.

**Reviewer #2 Comment 3**

*In a more general way, I do not find the logic of this experiment very convincing. The production of song is an extremely complex process that depends on the formation of connection between different brain nuclei throughout development. The manipulation that they do in this paper involves injecting a BDNF receptor agonist in the circuit of a fully formed circuit. The specific connections between the different brain nuclei have formed during the embryonic period and during song learning. However, activating BDNF receptor in an adult animals is just “messing” with up the connections that were already there. It is interesting that the song gets degraded in a more or less predictable manner, but calling this “species-specific features is not justified.*

**Response**

Our study tried to elucidate the transcriptional divergence of ZF and OF for generating species-specific “learned songs” (not song learning). Thus, we performed a set of experiments, including the song comparative analysis and RNA-seq, by using adult birds after the critical period of song learning. The pharmacological experiment was also performed at the adult stage.

We fully agree with the reviewer regarding the importance of considering the developmental process of neural circuits during the embryonic and song learning periods. In the previous version of the manuscript, we discussed the potential contribution of the AFP to promote species-specific song learning. We also discussed further the potential neural mechanism for species-specific song learning and production (page 21).

**Reviewer #2 Comment 4**

*In summary, I think that paper is well done, and is interesting, but I do not agree that the interpretation of the BNDF experiments should be toned down drastically – BDNF receptor activation clearly changes the song, but we cannot tell that it is changing with any type of specificity. Actually, I think that the paper would be stronger without the BNDF agonist injection experiments.*

**Response**

We appreciate the reviewer’s comments. We understand the reviewer’s concern in terms of overinterpreting the BDNF experiments on song change, which should not be attributed to species specificity. On the basis of this suggestion, the Results section was fully and carefully revised regarding the BDNF pharmacological experiments. In the revised version, we first explained the BDNF *trans*-effects on the putative downstream genes, which were examined by the RNA-seq experiment and were confirmed as the altered expression of the putative downstream genes after the manipulation (shown as new Fig 7A). Subsequently, we showed that song changes are caused not by the loss of specie-specificity but by post-manipulation degradation effects, which might be impinged by not only the BNDF *trans*-altered genes but also by other altered genes.

**Reviewer #2 Comment 5**

*More specific comments:*

*. Line 209: In particular, we found that GO categories related to neural functions associated with synapse transmission, somatodendritic compartment, and nervous system development were significantly enriched for RA trans-regulated genes.*

*I found this statement tautological –genes associated with “synapse transmission, somatodendritic compartment, and nervous system development” comprise a huge number of the genes expressed in neurons. Thus, it is expected that simply because of their sheer number, those genes are the ones whose pattern of expression are going to change.*

**Response**

We understand the reviewer’s point and agree with the tautological problem of GO terms (categories).On the basis of the reviewer’s suggestion, we have revised the examples of GO terms to show more specific neural functions, such as “presynapse, chemical synapse transmission, and neuron projection” (second paragraph on page 12).

**Reviewer #2 Comment 6**

*. Line 221 “ this prediction of BDNF as a trans-regulatory mediator in RA, we found two amino acid substitutions in BDNF between ZF and OF: Ser45Arg in prodomain and Thr143Met in NGF domain (Fig. 5D)”.*

*And Line 247: “In contrast, the expression level of BDNF mRNA in HVC had four significant correlations with acoustic and sequential song parameters in F1 hybrids [acoustics: syllable duration (r = −0.862, p =0.006) and entropy vibrance (r = −0.822, p = 0.012); sequence: motif (r = −0.762, p = 0.028) and repetition (r = 0.729, p =0.040), Pearson correlation] (Fig. 6C, D).”*

*I find this argument very confusing. The main type of measurements that they do are related to levels of gene expression. However, if in addition to changes in levels of expression, there are also changes in the sequence of the genes this would make it extremely difficult to separate which of these 2 factors is responsible for the behavioral changes that they observed.*

**Response**

As actual *trans*-acting ways driven by transcriptional regulatory alteration, the amino acid substitution and expression level change of *trans*-regulatory mediator genes could be considerable. Therefore, we checked the BDNF in terms of these two factors. As suggested by the reviewer, it is not easy to separate the effect level of the two factors. We realized that interspecies F_1_ hybrids could be a good model for evaluating the putative *trans*-regulatory effects mediated by the amino acid substitution or expression level because they generally show individual differences in both gene expression level and ASE ratio. Therefore, the expression level and ASE ratio values in F_1_ hybrids could be used for the correlational analyses against their individual behavioral phenotypes.

The Results section was revised to clarify the information described above (pages 13–14).

**Reviewer #3:**

**Reviewer #3 Comment 1**

*This paper uses F1 species hybrids to investigate how transcriptional regulatory divergence between species relates to the production of species-specific songs. I generally found the question to be interesting and well-addressed. I have a few concerns, primarily about the conclusions reached with the last experiments, and well as some more minor editorial comments. In general, there are a number of places where the manuscript needs more editing for clarity and readability.*

**Response**

We thank the reviewer for the interests shown in our study and for the many constructive comments, which helped us improve our manuscript. Following the reviewer’s suggestion, the manuscript was revised for language, clarify, and readability by a native English speaker.

**Reviewer #3 Comment 2**

*My main concern is with the conclusion that over-activation of BDNF eliminates species-specific acoustic and sequence traits. While there are clearly changes to ZF song with over-activation of BDNF, those changes are reminiscent of simplification of song structure and increases in repeats seen with a range of other manipulations where you lose syllable complexity/diversity and increase repetition. For example, the greater stereotypy and syllable degradation you see with lesioning LMAN/DLM in young birds, or changes with deafening or with different acoustic experience during development. While a number of measures of song move in the direction of the Owl Finch song, I’m not convinced that is the same as the song becoming more like OF sing vs. just becoming simpler and noisier. It’s also not clear why changes to BDNF level would make the song more like OF song, unless it was thought that the shared ancestor of OF and ZF had a song more similar to OFs, which isn’t clear based　on the songs of other grass finch species in the clade or something discussed here.*

**Response**

As suggested by the reviewer, the results from the BDNF pharmacological experiments should have been more carefully interpreted. As indicated by the reviewer, the song changes observed after BDNF pharmacological manipulation were most likely caused by song degradation rather than by species-specific song feature changes to resemble owl finch songs. Therefore, all related paragraphs in the Results and Discussion sections were fully revised. Moreover, information regarding owl finch song features were removed from previous Fig 7C and 7E (Fig 7D and 7F in the revised manuscript) to avoid misinterpretation.

**Reviewer #3 Comment 3**

*I am also surprised by the weak effect of learning shown in Figure 1. Because 15-25 phd is really late for removing the father, I wonder if this is why there is not a stronger effect of the cross tutoring?*

**Response**

Zebra finch chicks removed from their father by 15–25 phd usually learn tutor songs well in our laboratory. As described in the Materials and Methods (second paragraph on page 23), owl finch chicks were hand reared after hatching until they could feed themselves and totally deprived from hearing conspecific songs. Although we did not evaluate the precise effects of the timing of removal on chicks’ song acquisition, we speculate that song learning is more strictly regulated by genetic constraints between the two species.

**Reviewer #3 Comment 4**

*Drug infusion: 10 days is a long time for a permanently implanted microdialysis probes, were there any issues with gliosis? How was it “infused continuously during daytime”? Is it using the reservoir system? If so, Is there any data to demonstrate that there is still drug moving from the reservoir into the probe itself at the end of the day? For example, when was song recorded for the TTX controls? If it is late in the day, that could support that drug is continuing to be infused.*

**Response**

After BDNF pharmacological manipulation, brain samples were used for the RNA-seq experiment. Hence, this experiment did not provide information regarding gliosis. However,  microdialysis probes were carefully set outside the RA to avoid the song changes induced by physical cellular damage. We injected ~2.5 uL of 7,8-DHF into the outer reservoir of the microdialysis probes for three to four times daily (S8 Fig). Thus, microdialysis probes were not connected with the external reservoir. Owing to the use of a transparent polyimide tube as the outer reservoir of the microdialysis probes, we could easily see the remaining drug volume o and infusion speed.

This information was added to the Materials and Methods section of the revised manuscript (second paragraph on page 29).

**Reviewer #3 Comment 5**

*Minor comments*

*Line 42: analog not homologue*

**Response**

We thank the reviewer for this correction, the term has now been corrected.

**Reviewer #3 Comment 6**

*Line 47: eliminated species-specific acoustic and sequence traits is too strong.*

**Response**

Following the reviewer’s suggestion, we revised the wording as “disrupting the learned song structures of adult zebra finch songs at the acoustic and sequence levels” (page 2).

**Reviewer #3 Comment 7**

*Line 54-55: I’m not sure that ecology makes sense here. Not clear on what this is saying.*

**Response**

We replaced the word “ecology” by the term “habitat use.”

**Reviewer #3 Comment 8**

*Line 70: song system? Song pathway makes it sound especially linear.*

**Response**

On the basis of this suggestion, we replaced “song pathway” by “song system.”

**Reviewer #3 Comment 9**

*Line 80: refs are mostly about Area X*

**Response**

As indicated by the reviewer, two of three references (Refs. 25 and 26) reports different expression of FoxP2 and AR in Area X in different species. However, in Ref. 27, Asogwa et al. (2018) reported the inter- and intraspecies differences of mAChR2 expression in the HVC. To our knowledge, the report from Asogwa et al. (2018) is the first to show species-specific gene expression in a song nucleus (HVC) in the vocal motor pathway.

**Reviewer #3 Comment 10**

*Line 91-92: this sentence is difficult to follow, rephrase.*

**Response**

Following the reviewer’s suggestion, we revised the following sentence: “we assessed the total number of genes whose expression differences by divergence in *cis*- versus *trans*-transcriptional regulation between the two species.” (page 5)

**Reviewer #3 Comment 11**

*Line 92: define Gene Ontology.*

**Response**

Gene Ontology is defined as “a bioinformatics initiative to unify the representation of gene and gene function across all species.” We think that the expression “Gene Ontology” is a standard term in molecular biology experiments.

**Reviewer #3 Comment 12**

*Line 102: Is this really a concern, that lab rearing would eliminate species-specific song features? There needs to be better justification for this.*

**Response**

We agree with the reviewer’s comment: songbirds maintained their species-specific song features under laboratory-controlled housing and breeding environments. However, our colleagues working in different research areas would often ask about the learning effects on song development under non-natural condition. Therefore, we first clarified this possibility in the manuscript.

**Reviewer #3 Comment 13**

*Line 108: I’m not convinced that having somewhat overlapping values for each individual acoustic feature is an indication that there are not some physical constraints of peripheral vocal organs. Also, the sentence is confusing, I think it means to say that because the distribution ranges are overlapping, they can confirm there is not a physical constraint, but that it not how it is written on Line 109.*

**Response**

We thank the reviewer for pointing this out. On the basis of the reviewer’s suggestion, the sentence was revised to the following “We found that the range, but not the pattern, of each acoustic feature’s distribution overlapped between ZFs and OFs (3,000 syllables from n = 6 birds each and 500 syllables/bird) (**S1 Fig**), thus suggesting that the species differences in the syllable acoustics were not caused by physical species-specific constraints in the peripheral vocal organs.” (first paragraph on page 6)

**Reviewer #3 Comment 14**

*Line 116-120: It looks like the statistics were to compare within each tutoring condition (normal vs. cross-species). I’m not clear on what was done statistically to say that there are song tutoring effects on most of the song parameters. This would mean whether there is a difference between ZF tutored ZFs and OF tutored ZFs, correct? But none of those statistics are reported. The differences within the cross-tutored groups are convincing, and the main point of the figure, but as written this paragraph makes me wonder about the effects of tutoring. I think a statistical model with tutoring condition, species, and the interaction term would be useful here. It also seems like a lot of statistical tests (8 different song measures) to do with a very small sample size.*

**Response**

The effects of song tutoring on each song parameter were analyzed by comparing the *p*-values of differences in the acoustic features between conspecific and cross-species song tutored groups. For example, regarding entropy variance, conspecific-tutored ZF and OF showed a significant difference with *p*** < 0.01. By contrast, cross-species song tutored ZF and OF showed *p** < 0.05. Thus, we considered that entropy variance was affected by song tutoring contents.

On the basis of the reviewer’s suggestion, we added PCA by using the information of tutoring condition, species, and eight song parameters (motif, repetition, and six acoustic features). In line with our description, PCA showed that the songs of these two species are mainly developed by genetic constraints and are weakly affected by the song tutoring conditions (shown as new S1 Fig panel C).

This information was added to the revised Results section (first paragraph on page 7).

**Reviewer #3 Comment 15**

*Line 166-167 “we identified that over 75 and 10% of examined genes” I don’t understand what this means.*

**Response**

Following the reviewer’s suggestion, we revised the sentence as follows “we observed that over 75% and 10 % of the examined genes were expressed in both HVC and RA with either “conserved” or “ambiguous” regulation between ZF and OF, respectively (**Fig 4B**).” (first paragraph on page 10) .

**Reviewer #3 Comment 16**

*Line 183: what does “between a pair of eight F1 hybrids” mean?*

**Response**

It means “a cross-replicate comparison of ASE ratio using eight F1 hybrids”. The phrase was revised accordingly. (first paragraph on page 8)

**Reviewer #3 Comment 17**

*Line 240-243: This statement needs to be more clear. Birds were reared hearing both ZF and OF songs? Just single examples of each? But each bird acquired an individually-unique song?*

**Response**

On the basis of the reviewer’s suggestion, we revised the sentence as “F_1_ hybrids acquired individually unique songs with a wide range of ZF- and OF-biased features even though they were reared by having them listen to both ZF and OF songs as models” (second paragraph on page 10). Further information about the song tutoring condition of F_1_ hybrids was added to the Materials and Methods section (second paragraph in page 23).

**Reviewer #3 Comment 18**

*Line 245: I don’t think it’s “as a result”, delete this phrase.*

**Response**

Following the reviewer’s suggestion, we deleted the phrase.

**Reviewer #3 Comment 19**

*Line 249: Entropy variance? Not “vibrancy”*

**Response**

We thank the reviewer for pointing out the typo. This error has now been corrected.

**Reviewer #3 Comment 20**

*Line 258-261: The justification for looking at BDNF needs to be explained more clearly.*

**Response**

On the basis of the suggestions of Reviewers #2 and #3, the Result section was fully revised regarding the BDNF pharmacological experiments. It was also clarified that the main purpose of the experiments was to evaluate the potential *trans*-regulatory effects of BDNF on the downstream genes in RA in vivo (pages 16–17).

**Reviewer #3 Comment 21**

*Line 314: word missing, regulated ___ in? by?*

**Response**

We revised the sentence as follows “In principle, two alleles in the cells of an F_1_ hybrid are regulated in the same *trans*-regulatory environment.” (second paragraph on page 19)

**Reviewer #3 Comment 22**

*Line 342: axonal boutons, not buttons*

**Response**

We thank the reviewer for pointing out this typo. This error has now been corrected.

**Reviewer #3 Comment 23**

*Line 347-349: This sentence needs rephrasing*

**Response**

On the basis of the reviewer’s suggestion, we revised the sentence as “Species differences in BDNF expression in the song nuclei could shape the anatomical and molecular bases for the generation of species-specific learned song structure via the activity-dependent *trans*-regulation of the downstream genes.” (first paragraph on page 20)

**Reviewer #3 Comment 24**

*Line 361-363: Needs a better description of the AFP. The basal ganglia is homologous across vertebrates. The AFP is a specialized cortical-basal ganglia-thalamic loop*

**Response**

We revised the sentence as “The anterior forebrain pathway (AFP) is a cortico-basal ganglia-thalamocortical loop, which is a specific pathway for song learning during development and for vocal plasticity maintenance later in life.” (third paragraph on page 21)

**Reviewer #3 Comment 25**

*Line 369: I’m not sure that “motif structure” is a species specific trait of ZF songs. This seems to be referring to a very specific feature of motif structure, but that’s not clearly defined here.*

**Response**

Following the suggestion, we revised the phrase as “motif structure, a sequential trait commonly observed in ZF songs” (first paragraph on page 22).

**Reviewer #3 Comment 26**

*Line 402: OF chicks not checked.*

**Response**

We thank the reviewer for pointing out the typo. We corrected the error.

**Reviewer #3 Comment 27**

*Line 581: rephrase, was infused to one hemisphere of RA.*

**Response**

We revised the sentence as follows: “tetrodotoxin (TTX, 6–12 μM) was infused into the RA in a hemisphere, and a hemi-RA inactivation-induced song change was confirmed.” (second paragraph on page 29)

**Reviewer #3 Comment 28**

*Fig 2: Plots in 2B should be on the same scale for con and cross.*

**Response**

Based on this suggestion, we changed the graphs to show the same scale.

**Reviewer #3 Comment 29**

*Fig 6: Labels in legend are wrong relative to the figure (also out of order).*

**Response**

We thank the reviewer for pointing out the mislabeling. We corrected the figure labels in the figure legend.

**Reviewer #3 Comment 30**

In general, there needs to be more information in the Methods about the rearing of F1 hybrids and how they were tutored.

Response

As answered in our response to Comment 17, further information on the song tutoring conditions of F_1_ hybrids was added to the revised Materials and Methods section (second paragraph in page 23).
